# Multilineage lymphoid clonal hematopoiesis: a cross-sectional, correlative analysis of plasma cell dyscrasias and large granular lymphocytic leukemia

**DOI:** 10.1038/s41375-026-03071-5

**Published:** 2026-08-05

**Authors:** Carlos Bravo-Perez, Carmelo Gurnari, Arda Durmaz, Miguel Ruiz, Zachary Braunstein, Naomi Kawashima, Justin Jiang, Zachary Brady, Atsushi Marumo, Arooj Ahmed, Mark Orland, Aashray Mandala, Luca Guarnera, Matteo D’Addona, Serhan Unlu, Christopher Haddad, Kartik Lakhotiya, Praveena Thiagarajan, Megan Nakashima, Heesun J. Rogers, Jonathan E. Brammer, Valeria Visconte, Jaroslaw P. Maciejewski

**Affiliations:** 1 Department of Translational Hematology and Oncology Research, Taussig Cancer Institute, Cleveland Clinic, Cleveland, OH USA; 2 Department of Hematology and Medical Oncology, Hospital Universitario Morales Meseguer, University of Murcia, IMIB-Pascual Parrilla, CIBERER—Instituto de Salud Carlos III, Murcia, Spain; 3 Department of Biomedicine and Prevention, University of Rome Tor Vergata, Rome, Italy; 4 Division of Hematology, Department of Internal Medicine, The James Comprehensive Cancer Center, The Ohio State University, Columbus, OH USA; 5 Department of Internal Medicine, The Ohio State University, Columbus, OH USA; 6 Robert J. Tomsich Department of Pathology and Laboratory Medicine, Diagnostic Institute, Cleveland Clinic, Cleveland, OH USA

**Keywords:** Haematological cancer, Cancer genomics

## Abstract

Clonal expansions in large granular lymphocytic leukemia (LGLL) may arise in response to immune dysregulation in the context of other hematologic malignancies. This study aimed to investigate the co-occurrence of LGLL and plasma cell dyscrasias (PCDs) to assess its prevalence, features, and potential underlying mechanisms. We conducted a cross-sectional study involving 2064 PCD cases and 534 LGLL cases. Of the 534 LGLL cases, 20% co-occurred with PCD, while LGLL was present in 2% of the 2064 PCD cases. Among 117 patients with both conditions, PCDs were predominantly associated with IgM M-protein, while LGLL patients had increased NK-cell proliferation and fewer mutations in *STAT3/5B*. The co-occurrence was linked to a higher incidence of autoimmune conditions, B-cell neoplasms, and more severe immune-mediated cytopenias. In symptomatic PCD/LGLL patients with low plasma-cell infiltration, B-cell-targeted therapies outperformed immunosuppression, suggesting humoral involvement. Correlation with biological parameters revealed elevated interleukin-6 and inhibitory effects of sera of symptomatic PCD/LGLL patients on hematopoietic cells. Our findings suggest an association between PCD and LGLL, characterized by distinct features that may have significant implications for clinical management. These results highlight the importance of considering hematologic co-occurrences in clinical practice and warrant further investigation into the molecular mechanisms linking these conditions.

**Figure:**
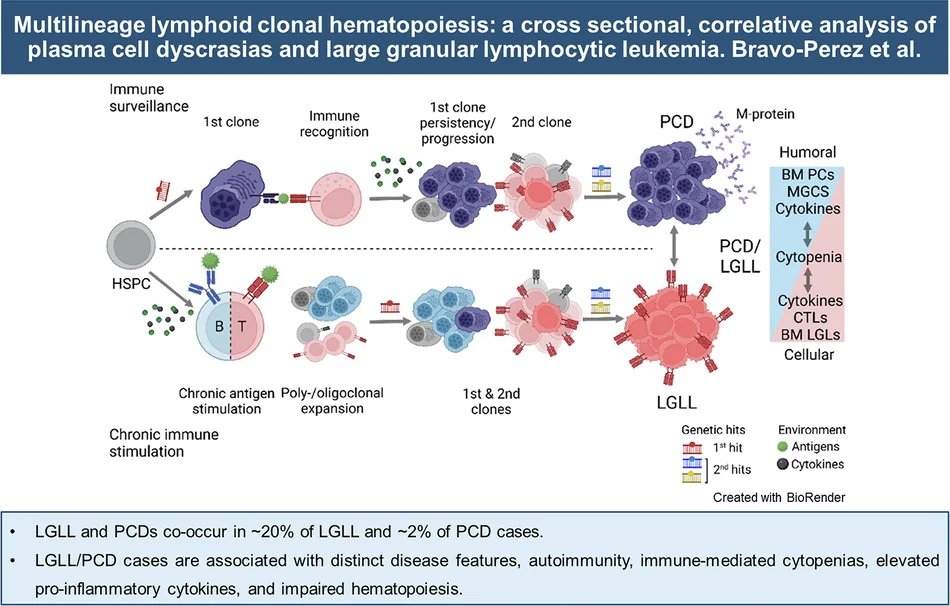

## Introduction

In the last decades, precursor cell clones with putative abnormalities for different blood malignancies have been appreciated, not only in otherwise healthy individuals but also in patients with various conditions, including a preexisting hematological malignancy (HM) [1, 2]. This is the case of the co-occurrence of large granular lymphocytic leukemia (LGLL) with B-cell lymphoid neoplasms/monoclonal B-cell lymphocytosis (BCN/MBL) [3–5], or with myeloid neoplasms/clonal hematopoiesis (MNs/CH) [6–8].

LGLL is a chronic lymphoproliferative disease defined by the clonal expansion of cytotoxic lymphocytes (CTLs) [9]. Mutations in *STAT3* and *STAT5B* are detected in 40-60% and 1% cases, respectively [10–13]. A majority of patients eventually develop severe cytopenia and require immunosuppressive therapy (IST) [14]. Evidence supports that clonal expansions in LGLL may develop from immune responses in the context of infectious, rheumatologic or neoplastic diseases [15]. Of special interest are the associations of LGLL with other HMs and their precursor states. These clinical associations constitute unique research frameworks to discern whether the LGLL clones are causes or consequences of the concomitant clonal entity, or which clone should be targeted in the case of cytopenia and the choice of therapy [15, 16].

While investigating the clinical features of LGLL, we have noticed the frequent presence of monoclonal gammopathy of uncertain significance (MGUS) and other plasma cell dyscrasias (PCDs). This co-occurrence has also been reported in some case series [17, 18]. However, the frequencies of PCDs in LGLL and vice versa are not known, nor has a mechanistic connection between these two conditions been established. Both PCDs and LGLL mostly occur in the elderly [19], suggesting epidemiologic association. Alternatively, the two entities might originate from an initial immune response against a common antigen, or T-LGLL clones may target atypical plasma cells or the M-protein in the context of a tumor surveillance reaction.

In this study, we aimed to assess the epidemiology of the association of PCDs and LGLL, and determine the clinico-molecular features and the impact of this polyclonopathy state in both the PCD and LGLL settings.

## Methods

Full details are available in Supplementary Methods.

### Study design and participants

We addressed the cross-sectional and genetic association study of PCDs and LGLL with a dual approach: (1) PCD-forward strategy: screening for LGLL in individuals with bone marrow (BM) evaluation and documented diagnosis of PCD from Cleveland Clinic (2005-2021); (2) LGLL-forward strategy: search of cases with M-protein assessment (MPA), confirmed by serum electrophoresis (SPEP) and/or serum free light chain (FLC) assay, in a large, retrospective cohort of 534 cases with LGLL from two clinical centers (1994-2024): (i) Cleveland Clinic (*n* = 388) and (ii) The Ohio State University Wexner Medical Center (*n* = 146).

Diagnosis of LGLL was made based on the presence of >4/6 criteria: (i) large granular lymphocytes (LGLs) > 0.40 × 10^9^/L in blood for more than 6 months; (ii) abnormal T or NK CTLs (CD2 + , CD56 + , CD57 + , CD28-); (iii) preferential usage of a TCRVβ family or a TRBC1/2 chain by flow cytometry; (iv) clonal TCR gene rearrangement by PCR; (v) *STAT3/5B* mutations; (vi) LGLL infiltration in the BM [9].

Under the designation of PCD, we included the following entities: (i) IgM PCD, comprising IgM MGUS, IgM lymphoplasmacytic lymphoma/Waldenström macroglobulinemia, and IgM myeloma; and (ii) non-IgM PCD (IgG, IgA, IgD or light chain PCD), including non-IgM MGUS, and non-IgM myeloma. Definitions of MGUS and other PCDs were done according to the 5^th^ WHO Classification of Hematolymphoid Tumors [20]. Furthermore, distinction between myeloma/Waldenström macroglobulinemia in smoldering (SMM/SWM) or active (MM/WM) stages was done as defined by disease international workshops [21, 22]. Cases with low M-protein levels ( < 1 g/L) or M-protein detected in at least 1 but not all SPEP performed, described elsewhere as small or transient M-proteins, respectively, were also considered for analysis [23].

### Data collection and laboratory studies

Clinical data collected comprised patient comorbidities, cytopenia of any type and severity, and immune-mediated cytopenias including peripheral autoimmune cytopenias (autoimmune hemolytic anemia [AIHA], autoimmune neutropenia [AIN], immune thrombocytopenia [ITP]) and BM failure syndromes (pure red cell aplasia [PRCA], aplastic anemia [AA]) [14, 24]. Severe cytopenia was defined consistent with established hematologic criteria as hemoglobin <80 g/L, absolute neutrophil count <0.50 × 10^9^/L, or platelet count <25 × 10^9^/L [25, 26].

Adverse disease-specific clinical outcomes in LGLL analyzed were initiation of IST, transfusion dependency, splenectomy, hematopoietic stem cell transplant, and death. Cytopenia response to treatment was evaluated using ECOG5998 criteria [27]. Adverse disease-specific clinical outcomes of patients diagnosed with PCD included the presence of CRAB signs of symptomatic myeloma/lymphoma, and other paraneoplastic manifestations attributable to the M-protein that in the absence of >10% BM plasma cell infiltration would be indicative of a monoclonal gammopathy of clinical significance (MGCS) [28].

Laboratory data at diagnosis comprised CBC, BM evaluation, and routine tests performed as part of the diagnostic workup of LGLL (LGL count, lymphocyte subset characterization by flow cytometry, TCR rearrangement*, STAT3/5B* mutational status) and PCDs (MPA, Ig levels, myeloma FISH, *IGH* rearrangement, *MYD88* mutational status). Serum levels of inflammatory cytokines were determined using quantitative multiplex bead assays in routine clinic laboratory.

### Next-generation sequencing

*STAT3/5B* mutational status was assessed by deep targeted DNA sequencing [29]. Next generation sequencing (NGS) performed in genomic DNA extracted from peripheral blood mononuclear cells was available in 285 LGLL patients with either targeted sequencing performed using a custom panel for detection of hematological neoplasm gene variants from TruSeq or Nextera platforms (Illumina, San Diego, CA) and/or whole exome sequencing (WES) performed by Novogene America (Sacramento, CA) as previously described [8, 30, 31]. We also screened this molecularly genotyped cohort for the presence of somatic variants in a list of 80 CH-related genes (Supplementary Table 1). The mutational threshold was set at a variant allele fraction (VAF) of 1%.

### Bone marrow cultures

Healthy donors undergoing harvest of BM hematopoietic stem and progenitor cells were used for the collection of surplus cells purged from the filtration system. Erythroid and granulocytic cultures for detection and quantification of hematopoietic progenitor cells in colony-forming unit (CFU) assays were established in methylcellulose-based media (MethoCult™, STEMCELL^TM^ Technologies, Vancouver, Canada) with addition of either patient serum or normal pooled serum from type AB healthy donor volunteers (MP Biochemicals, Solon, OH) at a final concentration of 20%. The count of erythroid and granulocytic CFUs was assessed on day 14.

### Statistical analysis

Categorical variables were presented as percentages and compared using Pearson’s chi-square and Fisher’s exact tests. Continuous variables were presented as mean and standard deviation (SD), or as median and interquartile range (IQR) if non-normally distributed, and compared using Student’s *t* test or nonparametric Mann–Whitney U test. Survival analysis between groups was done with the Log-Rank test. Associations between clinical data and survival outcomes were assessed with unadjusted (univariable) and adjusted (multivariable) Cox regression. Overall survival estimations were presented with 95%CI. Differences with a 2-tailed *p* values of less than 0.05 were considered statistically significant. Unsupervised clustering of serum cytokine profiles was performed to identify patterns of immune activation across the cohort. Details on data preprocessing, normalization, and clustering methodology are provided in the Supplementary Methods.

Statistical analysis and graphic representation was performed using GraphPad Prism v.9.4 (GraphPad Software Inc., San Diego, CA) and STATA v.16 (StataCorp LLC, College Station, TX), or R (R Core Team, Vienna, Austria) [32].

## Results

### Identification of cases with PCD/LGLL and two-sided prevalence estimates

**Fig. 1:**
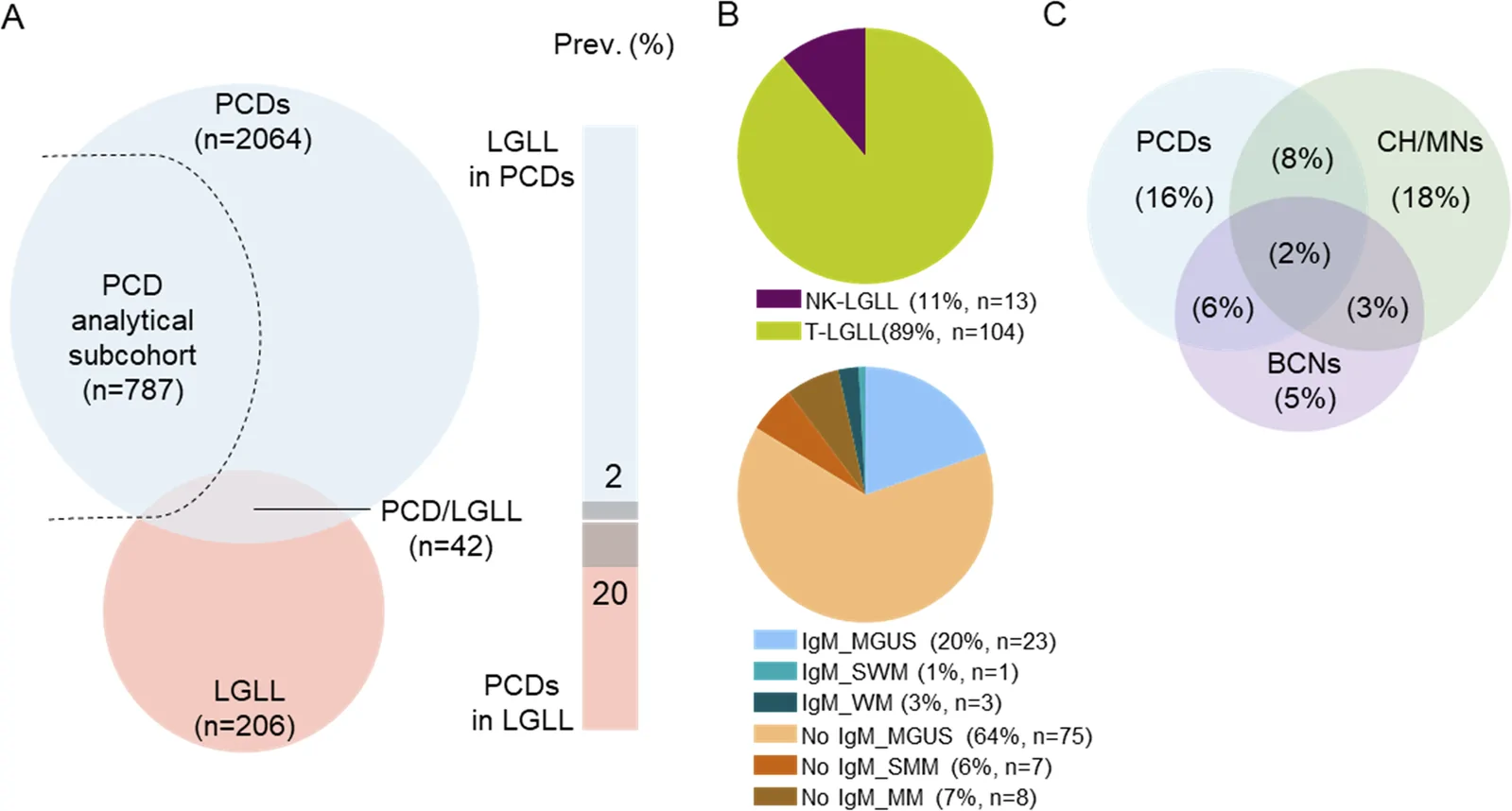
Summary of study design and cross-sectional frequency analyses. **A** Cross-sectional study. **B** Characterization of the PCD/LGLL cohort by disease subtype. **C** Concurrence of hematological malignancies and precursors states in LGLL cohort. BCNs B-cell lymphoid neoplasms, CH/MNs clonal hematopoiesis/myeloid neoplasms, LGLL large granular lymphocytic leukemia, MGUS monoclonal gammopathy of uncertain significance, SMM/SWM smoldering myeloma/Waldenström macroglobulinemia, MM/WM symptomatic multiple myeloma/Waldenström macroglobulinemia, PCD plasma cell dyscrasia.

Our strategy to build a cross-sectional and genetic association study was to apply a two-sided approach to the entire cohort: (1) screening for LGLL in individuals with PCDs; and (2) search of cases with PCDs in patients with LGLL. Characteristics of the PCD and LGLL cohorts are summarized in Table 1. Overall, we identified a total of 117 patients with concurrent diagnosis of PCD/LGLL: 42 from strategy no.1, and 75 from strategy no.2 (Supplementary Fig. 1A). First, we screened patients with PCDs for the presence of LGLL (Fig. 1A, Supplementary Fig. 1B). A total of 2064 (12%) of 17,847 PCD cases diagnosed at Cleveland Clinic (2005-2021) had MPA and BM examination. Ninety-nine (5%) of these cases had abnormal expansions of LGLs as per pathology assessment; and 42 met WHO LGLL diagnostic criteria, accounting for a 2% prevalence of LGLL in PCDs. A total of 206 (53%) of 388 cases with LGLL were diagnosed during the same period at the same institution and screened for PCDs by means of MPA and BM examination; the detection of these 42 cases accounted for a 20% prevalence of PCDs in LGLL. In addition, reverse screening of a total of 534 LGLL patients across two centers for the presence of PCDs identified additional 75 subjects with the PCD/LGLL dyad not included in the prior cross-sectional analysis.

**Table 1:** Characteristics of the PCD and LGLL cohorts.

PCD cohorts	Overall	Analytic
	*n* = 2064	*n* = 787
Gender (female), *n* (%)	891 (43.1)	325 (41.3)
Age at diagnosis (years), median (IQR)	70 (61–77)	70 (62–78)
Disease, *n* (%)		
Small/transient M protein^a^	48 (2.3)	18 (2.3)
MGUS	1229 (59.5)	435 (55.2)
SWM/SMM	191 (9.3)	79 (10.0)
WM/MM	445 (21.6)	168 (21.3)
Amyloidosis	151 (7.3)	87 (11.1)
M protein isotype, *n* (%)		
IgM	361 (17.5)	73 (9.3)
Non-IgM		
IgG	1136 (55.0)	456 (57.9)
IgA	315 (15.3)	132 (16.8)
Light chain	199 (9.6)	105 (13.3)
Other	53 (2.6)	21 (2.7)

LGLL cohorts	Cleveland clinic	Ohio State University
	*n* = 388	*n* = 146
Gender (female), *n* (%)	175 (45%)	77 (53%)
Age (years), median (IQR)	65 (56–74)	62 (53–71)
Autoimmunity, *n* (%)	165 (43%)	46 (32%)
Rheumatoid arthritis, *n* (%)	62 (16%)	29 (20%)
Disease, *n* (%)		
T-LGLL	364 (94%)	140 (96%)
NK-LGLL	24 (6%)	6 (4%)
LGL count (x10^9^/L), median (IQR)	1.26 (0.49–2.69)	1.39 (0.07–2.70)
Anemia, *n* (%)	223 (57%)	46 (32%)
Neutropenia, *n* (%)	161 (41%)	81 (55%)
Need of T-LGLL treatment, *n* (%)^b^	246 (63%)	99 (68%)
Follow-up (mo), median (IQR)	49 (16–105)	49 (47–52)
Mutational status, *n* (%)	*n* = 335	*n* = 146
*STAT3* mutation	119 (36%)	34 (23%)
*STAT5B* mutation	5 (1%)	0
MPA available	355 (91%)	59 (40%)

*IQR* interquartile range, *MGUS* monoclonal gammopathy of uncertain significance, *SWM/SMM* smoldering Waldenström macroglobulinemia/ smoldering myeloma, *WM* Waldenström macroglobulinemia, *MM* multiple myeloma, *PCD* plasma cell dyscrasia, *T-LGLL* T-cell LGL leukemia, *NK-LGLL* natural killer LGL leukemia, *MPA* monoclonal protein assessment.

^a^Small and transient M-proteins defined by low M-protein levels ( < 1 g/L) or M-protein detected in at least 1 but not all SPEP performed, respectively.

^b^Disease-specific systemic treatment, supportive treatment, therapeutic splenectomy.

### Clinical and molecular characterization of the PCD/LGLL dyad

Next, we investigated the clinico-molecular features of the 117 PCD/LGLL cases identified. Median age was 65 years (IQR 58–74); 54 (46%) patients were females. The M-protein isotype was IgG in 63 (54%) of 117 patients, IgM in 27 (24%), IgA in 10 (9%), light chains in 14 (12%) and oligoclonal in 4 (3%) patients. At diagnosis, 98 (84%) of 117 PCDs were MGUS, 8 (7%) had SMM/SWM, and 11 (9%) presented with active disease due to MM, WM or amyloidosis. The LGLLs were mostly of T-cell subtype (89% [104] of 117 cases); gain-of-function mutations in *STAT3*/*5B* were detected in 34 (30%) of 113 patients. Other concurrent B-cell and myeloid clonal disorders were found in 31 (27%) and in 29 (25%) of 117 patients, respectively (Fig. 1B, C).

**Fig. 2:**
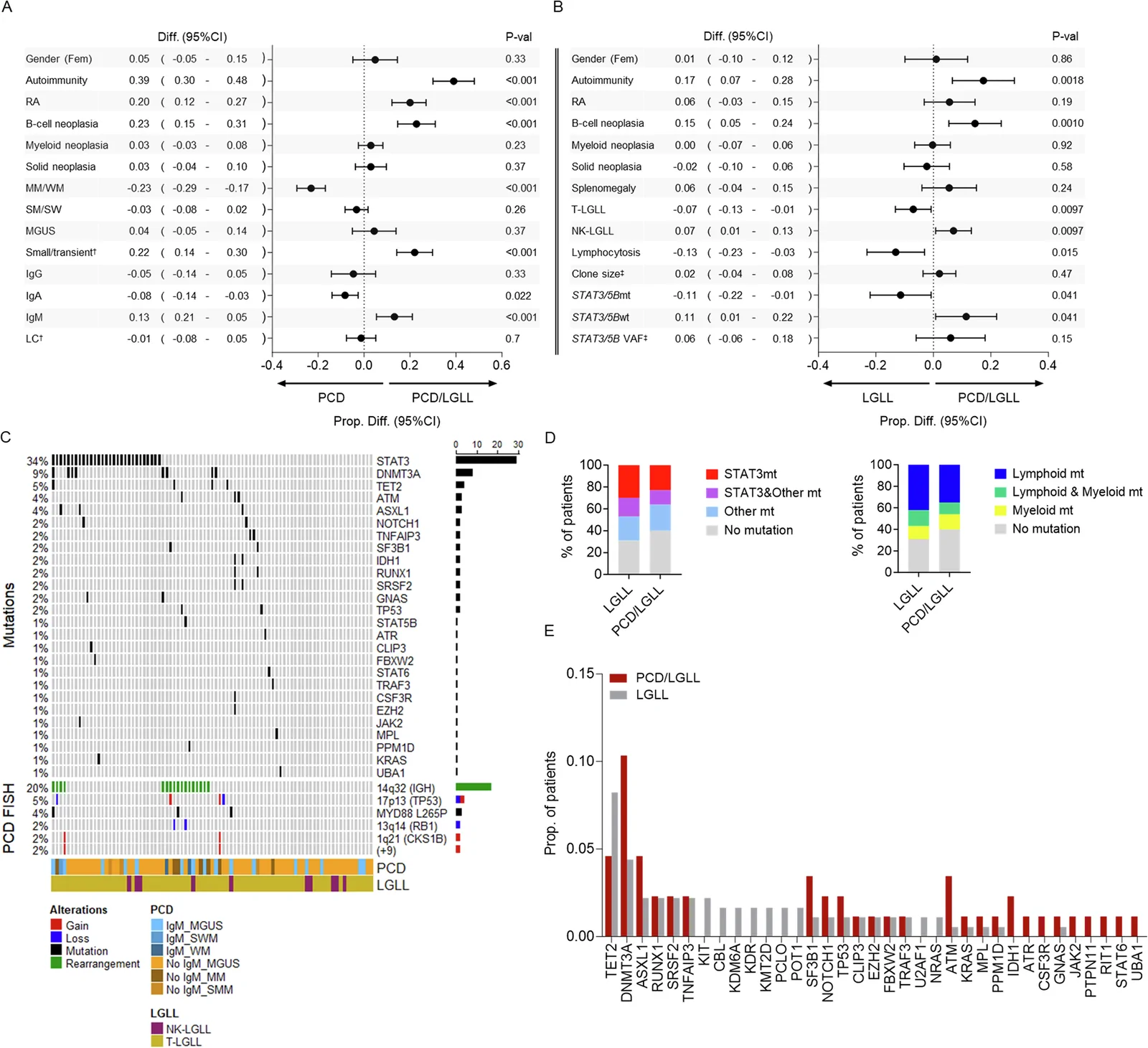
Clinical and molecular features of the PCD/LGLL dyad. **A**, **B** Comparison of PCD/LGLL vs. sole PCD and sole LGLL. Proportion differences and 95%CI are shown. **C** Mutational landscape and plasma cell cytogenetic abnormalities in PCD/LGLL cohort. **D**, **E** Frequency of clonal hematopoiesis-related mutations in PCD/LGLL and comparison with sole LGLL. LGLL large granular lymphocytic leukemia, MGUS monoclonal gammopathy of uncertain significance, SMM/SWM smoldering myeloma/Waldenström macroglobulinemia, MM/WM symptomatic multiple myeloma/Waldenström macroglobulinemia, PCD plasma cell dyscrasia.

We then compared the features of the PCD/LGLL cases vs. those of patients with only PCD or LGLL (Fig. 2A**)**. Overall, the PCD/LGLL dyad was more frequently associated with concurrent autoimmune conditions (57% vs. 15% in PCDs and 40% in LGLL, *p* < 0.001 and *p* = 0.0018, respectively), as well as B-cell lymphoid neoplasms (27% vs. 5% in PCDs and 13% in LGLL, *p* < 0.001 and *p* = 0.001, respectively). When compared to the PCD cohort, PCD/LGLL cases presented a younger age of diagnosis (median 65 vs. 70 years, *p* < 0.001), and had a higher frequency of IgM disease (22% vs. 9%, *p* < 0.001). Small or transient paraproteinemias were significantly enriched in patients with PCD/LGLL (24% vs. 2% in PCDs, *p* < 0.001). As compared to the LGLL cohort, cases with PCD/LGLL had a higher rate of LGL proliferations of the NK-cell subtype (11% vs. 4%, *p* = 0.0097). Indeed, among NK-LGLL cases in our cohort, a concurrent PCD was detected in up to 13 (54%) of 24 patients. In addition, albeit having a similar degree of LGL clonal expansion, PCD/LGLL patients presented less frequently with absolute lymphocytosis (27% vs. 40% in LGLL, *p* = 0.015). Mutations in *STAT3/5B* were less frequent in PCD/LGLL: 34 (30%) of 114 vs. 92 (41%) of 230 in only LGLL patients, *p* = 0.041. The frequency of mutations in other CH-related genes did not differ between PCD/LGLL dyad and LGLL patients (Fig. 2B–D, Supplementary Tables 1 and 2).

### Analysis of the risk of cytopenia in PCD/LGLL by type and severity

**Fig. 3:**
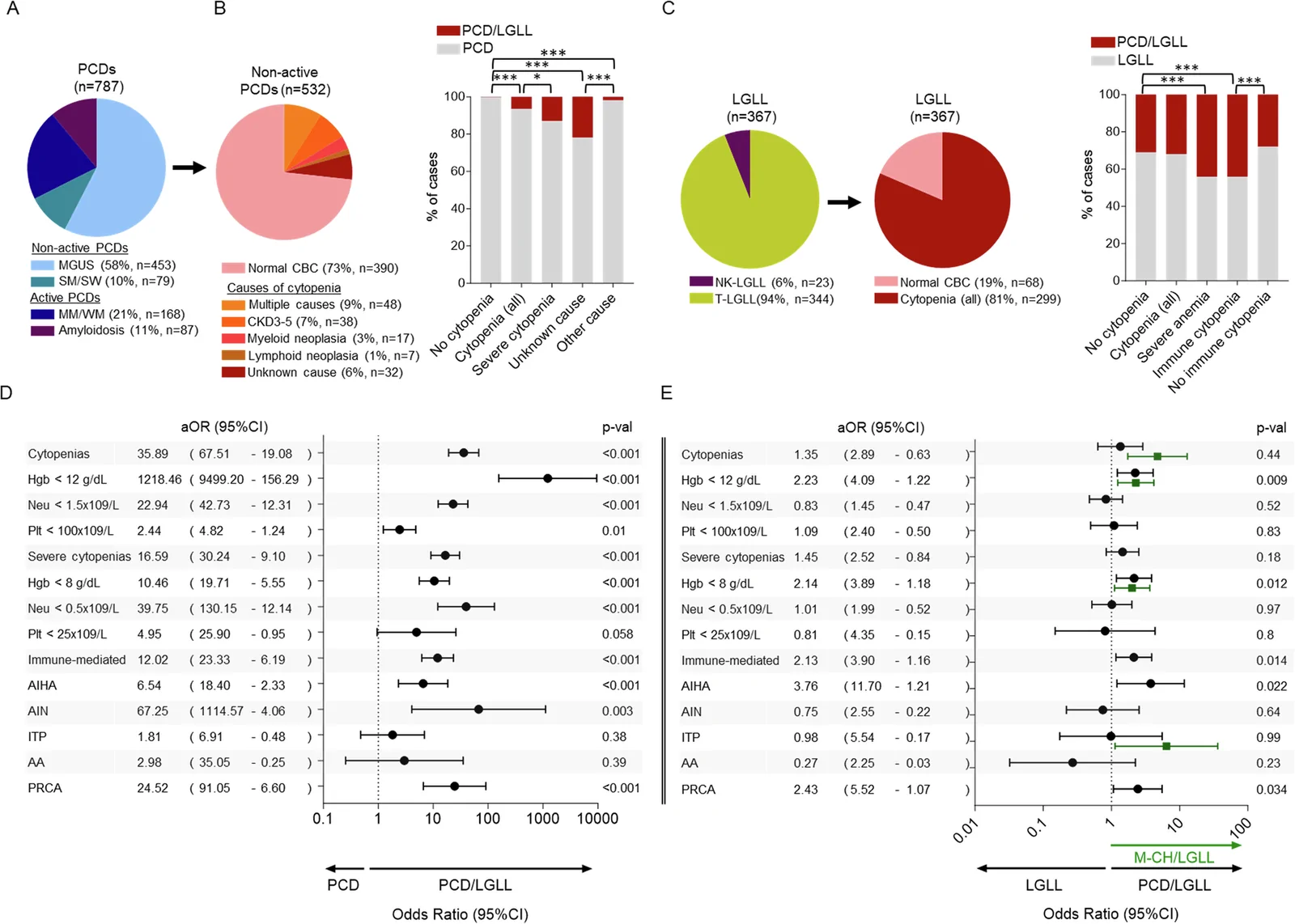
Clinical impact of the PCD/LGLL dyad on the risk of developing cytopenia. **A**, **B** Risk and type of cytopenia attributed to LGLL in patients with PCDs. **C** Risk and type of cytopenia attributed to PCD in patients with LGLL. **D** Multivariable logistic regression analysis on the risk of cytopenia in PCD caused by LGLL by lineage and severity. Odds ratios adjusted by gender, age and PCD clinical risk score. **E** Multivariable logistic regression analysis on the risk of cytopenia in LGLL caused by PCD by lineage and severity. Odds ratios adjusted by gender, age, *STAT3/5B* and other CH-related genes mutational status. The green intervals correspond to significant contributions of myeloid CH-related mutations. */*p* < 0.05, **/*p* < 0.01, ***/*p* < 0.001. AA aplastic anemia, AIHA autoimmune hemolytic anemia, AIN autoimmune neutropenia, aOR adjusted odds ratio, Hgb hemoglobin, ITP immune thrombocytopenia, LGLL large granular lymphocyte leukemia, MGUS monoclonal gammopathy of uncertain significance, SMM/SWM smoldering myeloma/Waldenström macroglobulinemia, MM/WM symptomatic multiple myeloma/Waldenström macroglobulinemia, M-CH myeloid clonal hematopoiesis, Neu neutrophils, PCD plasma cell dyscrasia, Plt platelets, PRCA pure red cell aplasia.

Next, we focused our analysis on the development of cytopenia, investigating whether LGLL could be a contributing factor in patients with PCD. We randomly selected an analytical subcohort of 787 individuals from 2064 PCDs for an in-depth investigation into the frequency and underlying causes of cytopenia (Fig. 3A, B). As in the original cohort, the prevalence of LGLL in this subsample was 2%. A total of 532 (68%) of 787 patients had non-active PCDs (i.e., MGUS, SMM/SWM), and 142 (27%) of 532 had cytopenia of any lineage and grade that could not be attributed to the BM plasma cell infiltration. Sixty-one (43%) of 142 cytopenia cases were severe. Screening for comorbid conditions explaining the cytopenia left 32 (23%) of 142 cases presumed to be immune-mediated and/or idiopathic. We found a significant enrichment of LGLL diagnoses in non-active PCDs with cytopenia (6% vs. 0.3% in cases without CBC alterations, *p* < 0.001). This frequency was even higher when considering only cases with severe cytopenia (13% vs. 1% in mild-moderate cytopenia, *p* = 0.011), and with idiopathic cytopenia (22% vs. 2% in cases with cytopenia of other causes, *p* < 0.001).

We then investigated the potential pathogenetic role of PCDs on the development of cytopenia in the LGLL setting (Fig. 3C). A total of 299 (81%) of 367 LGLL patients developed cytopenia of any lineage and grade; 165 (55%) of 299 cases had severe cytopenia. Among patients with PCD/LGLL, 95 (81%) of 117 patients were cytopenic; 59 (62%) of 95 had severe cytopenia. Eleven (9%) of 117 patients were primarily diagnosed with active MM/lymphoma due to >10% atypical plasma cell infiltration of the BM and either classical CRAB symptoms (*n* = 9), and/or amyloidosis or other active disease-defining paraneoplastic manifestation attributable to the M-protein (*n* = 4). After excluding these 11 PCD/LGLL patients, we found that the presence of a non-active PCD did not additionally increase the already elevated risk of cytopenia in LGLL when considering any lineage and severity. However, evaluation of the type and grade of the cytopenia revealed that, as compared with patients with sole LGLL, there was a significant enrichment of PCDs in LGLL cases with anemia (67% vs. 56%, *p* = 0.054), severe anemia (34% vs. 22%, *p* = 0.014) and immune-mediated cytopenia including both antibody-mediated peripheral cytopenia and immune BM failure syndromes (31% vs. 19%, *p* = 0.011).

Multivariable analyses confirmed that in patients with non-active PCDs, the presence of LGLL implied a higher risk of cytopenia (adjusted odds ratio [aOR] = 35.9, 95%CI: 19.1–67.5, *p* < 0.001) (Fig. 3D, Supplementary Table 3). We also validated that in patients with LGLL, the presence of myeloid CH-related mutations but not PCDs implied an increased risk of cytopenia (aOR = 4.7, 95%CI: 1.7–12.9, *p* = 0.002); while the presence of PCDs conferred an independent risk of anemia (aOR = 2.2, 95%CI 1.2–4.1, *p* = 0.009) (Fig. 3E, Supplementary Table 4). Evaluation of the type of cytopenia also confirmed that immune-mediated cytopenias were specifically enriched by the concurrence of PCD/LGLL in both the PCD and LGLL settings (vs. PCDs: aOR = 12.0, 95%CI: 6.2–23.3, *p* < 0.001; vs. LGLL: aOR = 2.1, 95%CI: 1.2–3.9, *p* = 0.014). The highest risks were found for the development of AIHA (vs. PCDs: aOR = 6.5, 95%CI: 2.3–18.4, *p* < 0.001; vs. LGLL: aOR = 3.8, 95%CI: 1.2–11.7, *p* = 0.022) and PRCA (vs. PCDs: aOR = 24.5, 95%CI: 6.6–91.0, *p* < 0.001; vs. LGLL: aOR = 2.4, 95%CI: 1.1–5.5, *p* = 0.034).

### Analysis of disease-specific outcomes in patients with PCD/LGLL

**Fig. 4:**
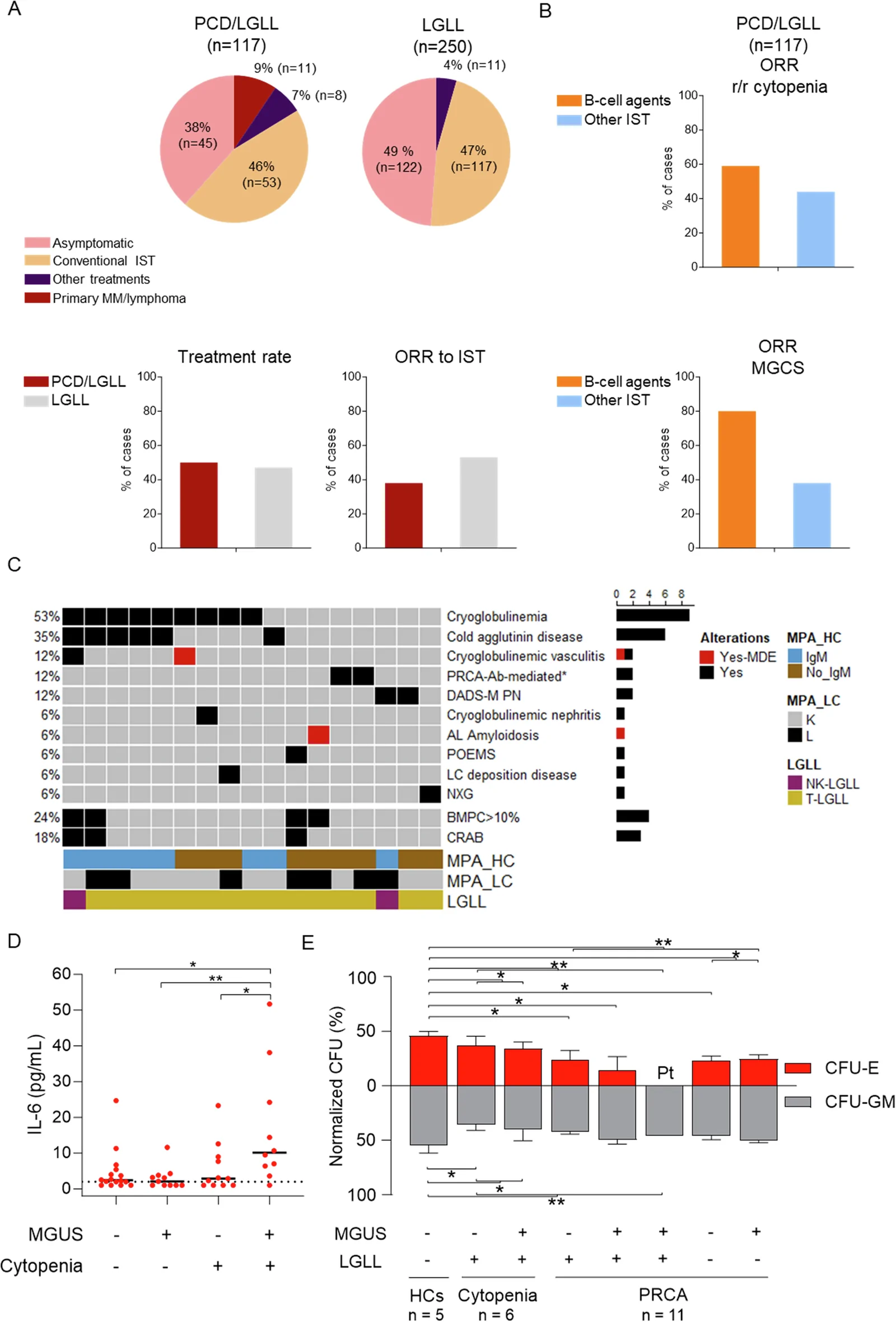
Clinical impact of the PCD/LGLL dyad attributable to the M protein: monoclonal gammopathies of clinical relevance in LGLL. **A** Treatment rate and ORR in PCD/LGLL vs sole LGLL. **B** Overall response rate of refractory cytopenias and paraneoplastic MGCS based on the IST therapy. **C** Description of MGCS syndromes in the LGLL cohort. In “PRCA-Ab-mediated”, the asterisk denotes that these cases of PRCA qualified as MGCS based on clinical assessment, specifically because they were r/r to IST and showed complete and durable responses to B-cell-directed agents. **D** Serum levels of IL-6 in LGLL patients with/without (w/wo) cytopenia, w/wo PCD (*n* = 47). **E** CFU assay of bone marrow cultures with sera of patients with LGLL and PRCA (w/o PCD), LGLL and other cytopenia (w/o PCD) and PRCA but no LGLL (w/wo PCDs) (*n* = 17). Pooled serum from AB group healthy individuals was used as reference (*n* = 5). Pt denotes a patient with PRCA with complete and durable response to bortezomib. */*p* < 0.05, **/ *p* < 0.01, ***/*p* < 0.001. BMPC bone marrow plasma cells, CFU colony-forming unit, DADS-M PN distal acquired demyelinating symmetric polyneuropathy associated with monoclonal gammopathy, HC healthy control, IST immunosuppressive therapy, LGLL large granular lymphocytic leukemia, MGCS monoclonal gammopathy of clinical significance, MGUS monoclonal gammopathy of uncertain significance, MPA M-protein assessment, MPA_HC heavy chain, MPA_LC light chain, NXG necrobiotic xanthogranuloma, ORR overall response rate, PCD plasma cell dyscrasia, POEMS polyneuropathy, organ enlargement, endocrine abnormalities, monoclonal protein, and skin changes, PRCA pure red cell aplasia, r/r relapsed/refractory, Yes-MDE presence of a paraneoplastic manifestation that constitutes a myeloma-defining event.

A total of 72 (62%) of 117 PCD/LGLL patients received specific therapy (Fig. 4A). Except for 11 (9%) patients treated with frontline immuno-chemotherapy because of active MM/lymphoma, 53 (50%) of the remaining 106 patients with non-active PCD received various forms of IST commonly used for cytopenia in LGLL. We focused our analysis on LGLL-specific outcomes. The rate of symptomatic LGLL requiring therapy was similar between patients with and without concurrent PCD (Supplementary Table 5). However, patients with PCD/LGLL had a lower ORR to frontline IST (38% vs. 53% in only LGLL, *p* = 0.065). Mean number of treatment lines per patient was 2 (IQR: 1–3), without differences between PCD/LGLL and LGLL patients. Among PCD/LGLL, the drug-specific ORR across all lines were 50% for cyclophosphamide, 41% for cyclosporine, and 35% for methotrexate.

Based on PCD/LGLL concurrence, 12 patients who were refractory to at least one line of conventional IST received B-cell-directed agents, which demonstrated an ORR comparable to that of IST used in the same setting of refractory disease (59% vs. 44%, *p* = 0.27) (Fig. 4B). The type of cytopenia in most of these cases was presumed to be immune-mediated, supporting that the concurrent PCD, despite a low tumoral burden, could be of clinical significance via humoral mechanisms. Retrospective clinical chart review identified 17 (15%) of 117 PCD/LGLL patients with paraneoplastic manifestations potentially attributable to the M-protein as per clinical and/or pathology assessment. Four (18%) of 17 patients had >10% BM plasma cell infiltration and therefore met criteria for active MM/lymphoma. As for the remaining 13 (76%) of 17 patients, the development of these complications without relevant plasma cell involvement qualified for a monoclonal gammopathy of clinical significance (MGCS, Fig. 4B, C). Twelve (92%) of 13 MGCS patients had cytopenia. Hematological manifestations were the most frequent form of MGCS (77%, 10/13 patients): 4 (31%) cases had cryoglobulinemia; 4 (31%) had cold agglutin disease (3 with concurrent cryoglobulinemia), and two (15%) cases had PRCA refractory to frontline IST that however showed complete and durable responses to B-cell-directed agents. Indeed, in patients with PCD/LGLL and probable MGCS, the ORR across all lines of treatment was superior for B-cell agents than for IST (80% vs. 38% for IST, *p* = 0.090), supporting that the clinical manifestation requiring therapy in this subgroup of patients might be caused by the humoral mechanisms rather than by the LGLL clone.

Median overall survival of the PCD/LGLL cohort was 129 months. In univariable and multivariable analysis, the probability of survival of PCD/LGLL patients was not different from that of cases with only PCD or LGLL (Supplementary Fig. 2, Supplemental Tables 5, 6).

### Disease-relevant inflammatory cytokines and effect on hematopoiesis

It has been suggested that abnormal cytokine signaling plays a key role in autoreactive conditions and lymphoid malignancies including LGLL and PCDs, also in the development of cytopenias [1, 33].

To explore whether distinct immune activation signatures could be identified, we performed unsupervised clustering of serum cytokine profiles in 22 LGLL patients with integrated genomic data (Supplementary Table 7). This analysis revealed two major groups, characterized by differential inflammatory activity: a pro-inflammatory cluster (cluster 1), which was enriched for patients with plasma-cell/ B-cell-related conditions, autoimmunity, and immune-mediated cytopenias, and a lower-level inflammatory cluster (cluster 2), which was enriched for *STAT3/5B*-mutant cases (Supplemental Fig. 3).

Within the pro-inflammatory cluster, interleukin-6 (IL-6) emerged as the most prominently elevated cytokine and was associated with the presence and severity of cytopenias. Extended analysis of serum IL-6 in 47 LGLL patients revealed that 32 (68%) had elevated levels. Importantly, IL-6 peaked in patients with PCD/LGLL and cytopenia (median 10.1 pg/mL vs. 2.9 pg/mL in patients with sole LGLL and cytopenia vs. 2.1 pg/mL in patients with asymptomatic PCD/LGLL vs. 2.5 pg/mL in patients with asymptomatic LGLL, *p* = 0.025). Furthermore, among patients with PCD/LGLL, cases with high IL-6 and immune-mediated cytopenias were significantly enriched (80% vs. 27% in only LGLL, *p* = 0.030). Specifically, there was an overrepresentation of cases with PRCA (40% vs. 9% in only LGLL) (Fig. 4D, Supplementary Fig. 4). We then performed CFU assays using healthy donor BM cells incubated with patient sera (Fig. 4E). As compared with controls, the sera of patients with LGLL had a negative effect on both the formation of erythroid (CFU-E median: 34.6% [IQR: 29.1–40.1%], vs. 45.8% [IQR: 41.7–49.1%] in controls, *p* = 0.023) and of granulocytic CFUs (CFU-GM median: 38.5% [IQR: 29.4–43.4%] vs. 49.6% [IQR: 49.1–62.2%] in controls, *p* = 0.011). Conversely, the sera of patients with PRCA selectively inhibited CFU-E (median: 21.9% [IQR: 19.6-27.2%] vs. 45.8% [IQR: 41.7–49.1%] in controls, *p* = 0.0018). The sera of patients with PCD/LGLL and PRCA yielded the lowest CFU-E counts (median: 19.6% [0–22.9%] vs. 20.4% [17.2–33.5%] in sole LGLL and PRCA, vs. 33.5% [27.4–40.1%] in PCD/LGLL with other cytopenia, vs. 35.6% [29.1–45.8%] in sole LGLL with other cytopenia, vs. 45.8% [IQR: 41.7–49.1%] in controls, *p* = 0.0056). Among the patients with PRCA and PCD/LGLL analyzed, we included one patient with refractory PRCA that showed complete and durable response to the B-cell agent bortezomib as previously described by our group [24]. Interestingly, the serum of this patient completely abolished CFU-E growth. The rest of sera from PRCA patients, including a case refractory to bortezomib, only produced partial CFU-E inhibitions.

## Discussion

Associations between seemingly unrelated entities often reveal important lessons of nature, and have the potential of guiding clinical decisions. We have performed a systematic, cross-sectional and two-sided analysis on the prevalence of LGLL in PCDs and vice versa, and accrued the largest series of PCD/LGLL patients to investigate the relationship between these two entities with a focus on the development of cytopenia [2]. Our study yields several clinically relevant conclusions. In the PCD setting, the estimated prevalence of LGLL was 2%. However, it is of note that this frequency was higher when considering cases with cytopenia not attributable to plasma cell BM infiltration or other easily discernable causes. Therefore, in patients with MGUS and unexplained cytopenia, LGLL diagnostic workup might be considered. In the reverse LGLL setting, a concurrent PCD was identified in 20% of patients, a figure that can be as high as 40-50% in cases with NK-LGLL. Thus, screening for PCDs is advisable in all LGLL cases at diagnosis and/or during follow-up. Previously, we described that myeloid CH-related mutations confer a greater risk of cytopenia in LGLL patients [8]. In this study, we show that PCDs imply a higher risk of anemia and immune-mediated cytopenia in LGLL. Our findings suggest that even “apparently non-active” PCD might be of hematological clinical significance through humoral mechanisms, justifying the term “hematological MGCS”. At a therapeutic level, our retrospective analysis on the response of cytopenias to a variety of agents suggests acceptable response rates using B-cell-directed drugs in these cases. Thus, in cytopenic PCD/LGLL patients refractory to conventional IST, in whom symptomatic LGLL and MGUS is presumed, the alternative of asymptomatic LGLL with an active PCD due to progression to MM/lymphoma or a MGCS may be possible. Especially, if there is a high suspicion of a MGCS as per pathology assessment or by clinical presentation (e.g. in patients with cryoglobulinemia and/or cold-agglutin disease), empirical treatment with B-cell agents could be considered.

Despite the primary clinical focus of our study, correlative analyses inspired stipulations as the pathogenesis of PCD/LGLL. Both PCD and LGLL are diagnosed at a median age ≥60 years. However, the younger age at PCD diagnosis of the patients with the dyad suggested that LGLL might either accelerate PCD detection or stem from a shared underlying pathogenic mechanism. Notwithstanding the possibility of lead-time bias, the significant association of PCD/LGLL with autoimmune disorders and B-cell neoplasms supports chronic antigenic stimulation as a strong pathogenetic factor driving low-level clonal lymphoid expansions [1]. Both LGLL and PCD are associated with rheumatologic diseases. Nevertheless, the burden of autoimmunity is substantially higher in LGLL than in PCDs [14, 34–36]. The tenfold higher frequency of PCDs in patients with LGLL, compared to that of LGLL in patients with PCDs, reflects the directionality of this relationship.

The chronic immune stimulation hypothesis is also supported by the evidence of increased inflammatory cytokines in patients’ sera. Cytokine clustering further refined this model by identifying two groups with distinct secretory profiles. The pro-inflammatory cluster, characterized by high IL-6 levels and systemic immune dysregulation, was enriched for B-cell/plasma-cell conditions, consistent with a cytokine-driven pathogenic LGLL subset. In contrast, the cluster with lower levels of inflammatory markers showed a higher frequency of *STAT3/5B* mutations, suggesting a more mutation-driven mechanism.

Importantly, despite the limited sample size of this exploratory analysis, which precludes robust conclusions regarding specific genotypes and does not allow direct comparison between NK- vs. T-cell LGLL subsets, our findings suggest that distinct cytokine profiles may reflect different pathogenic routes that nevertheless converge on activation of the IL-6/JAK/STAT3 axis through either cytokine-driven or mutation-driven mechanisms. Patients with PCD/LGLL and cytopenia had the highest values of serum IL-6. The transcription factor *STAT3*, which is activated by mutations in 40% of LGLL cases, is physiologically induced by IL-6 after binding to its receptor. Previous studies have also shown increased levels of IL-6 in LGLL, and support the relationship between cytokine release, clonal T-cell expansions, and disease-related immune phenomena [37–40]. The IL-6/JAK/STAT axis is also believed to be important for the development of PCD, since serum levels of IL-6 are increased in PCDs and correlate with prognosis [41, 42]. Proinflammatory cytokines have also been shown to have suppressive effects on hematopoiesis in different disease contexts, including rheumatoid diseases, PRCA, or HMs [33]. Abnormal cytokine signaling could therefore not only contribute to lymphoid expansions resulting in clonality, but also to the development of cytopenia during active disease stages.

Evidence supporting the tumor immunosurveillance hypothesis includes the frequent demonstration of oligoclonal expansions of terminal effector CTLs in patients with PCDs [43–45]. Recent studies using single-cell RNA/TCRαβ sequencing also show that hyperexpanded T-cell clones reactive to PCDs and T-LGLL clonotypes upregulate common gene sets related to T-cell cytotoxicity, activation, and exhaustion [43, 46]. In addition, in a myeloma patient with CTL expansion, reactivity of the T-cell clone against the autologous Ig idiotype was suggested [47]. Altogether, it is therefore conceivable that the hypotheses discussed above may not be mutually exclusive. Age-related decline in the immune response paired with chronic immune stimulation might first constitute a fertile soil for T- and B-cell lymphoproliferations. Once established, any or both the two clones may eventually induce or attract their counterpart, either by means of common growth signals, or as the result of a pathologic overcompensation of the immune system to fight a persistent/progressive clone. Similar dynamics may be observed in the co-occurrence of LGLL with other hematologic clonal entities such as MNs/CH or BCN/MBL [3–5, 8].

In conclusion, our study reveals novel associations between PCDs and LGLL that may have hematological clinical significance. These findings suggest alternative treatment strategies for cytopenias in PCDs and LGLL, providing a rationale for further investigations into the dynamics and crosstalk between hematologic clonal conditions.

## Supplementary information

Supplemental File

Supplemental Tables

## Data Availability

Additional information not present within the main text or in the supplemental material is available from the corresponding author upon reasonable request.
