# Supplementary material for: Multilineage lymphoid clonal hematopoiesis: a cross-sectional, correlative analysis of plasma cell dyscrasias and large granular lymphocytic leukemia
Source: Leukemia. 2026 Aug 5;40(11):2406–15. doi: 10.1038/s41375-026-03071-5 (PMC13713562; doi:10.1038/s41375-026-03071-5)
Supplement: Supplementary file 1 [file 41375_2026_3071_MOESM1_ESM.pdf]

Supplemental Material

Multilineage lymphoid clonal hematopoiesis: a cross-sectional, correlative analysis of plasma cell dyscrasias and large granular lymphocytic leukemia

Bravo-Perez et al.

Table of contents

Supplemental Methods 2
Study design and patients 2
Next generation sequencing 3
Whole-exome sequencing 3
Somatic variants filtering 4
Bone marrow cultures 4
Ethical approval 4
Statistical analysis 4
Supplemental Tables (table headings) 6
Supplemental Figures 7
Supplemental Figure 1 7
Supplemental Figure 2 8
Supplemental Figure 3 9
References 10

Supplementary Methods

Study design and patients

We addressed this cross-sectional and genetic association study with a dual approach: 1) PCD-forward strategy: screening for LGLL in individuals with bone marrow (BM) evaluation and documented diagnosis of PCD, collected from the Pathology archives of Cleveland Clinic (Jan 2005 to Dec 2021). 2) LGLL-forward strategy: search of cases with M protein, confirmed by serum electrophoresis (SPEP) and/or serum free light chain assay, in a large, retrospective cohort of 534 cases with LGLL from two tertiary institutions (Jan 1994 to Dec 2024): i) Cleveland Clinic (n=388) and ii) The Ohio State University Wexner Medical Center (n=146). Diagnosis of LGLL required the presence of >4/6 criteria: i) LGLs (>0.40 × 109/L) in blood for more than 6 months; (ii) abnormal cytotoxic T or NK lymphocytes expressing CD2, CD56 and CD57 and lacking CD28; (iii) preferential usage of a T-cell receptor (TCR) Vb family by flow cytometry; (iv) TCR gene rearrangement by PCR; (v) STAT3/5B mutation; (vi) LGLL infiltration of the BM (1).

Under the designation of PCD, we included the following entities: (i) IgM PCD, comprising IgM MGUS, IgM lymphoplasmacytic lymphoma/Waldenström macroglobulinemia, and IgM myeloma; and (ii) non-IgM PCD (IgG, IgA, IgD or free light chain [FLC] PCD), including non-IgM MGUS, and non-IgM myeloma. Definition of MGUS, as well as the other PCDs was done according to the 5th Edition of WHO Classification of Haematolymphoid Tumours (2). MGUS was defined by the presence of M-protein in serum (<30 g/L of Ig, or increased FLC with abnormal FCL ratio) or in urine (<0.5 g/24 hours), BM clonal plasma cells or lymphoplasmocytes <10%, and the absence of end-organ damage. Additionally, for Waldenström macroglobulinemia and myeloma, we further divided these entities into smoldering (i.e., SWM/SMM) and active disease (i.e., WM/MM) depending on the absence or presence of end-organ damage, as defined by the international workshops on WM (IWWM)(3) and myeloma (IMWG)(4). Cases with low M-protein levels (<1 g/L) or M-protein detected in at least 1 but not all SPEP performed, described elsewhere as small or transient M-proteins, respectively, were also considered for analysis (5).

Clinical data collected comprised patient demographics, comorbidities, associated autoimmune disorders and neoplasms, cytopenia of any lineage and severity, and immune-mediated cytopenias including peripheral autoimmune cytopenias (autoimmune hemolytic anemia [AIHA], autoimmune neutropenia [AIN], immune thrombocytopenia [ITP]) and BM failure syndromes (pure red cell aplasia [PRCA], aplastic anemia [AA]) (2, 6). Severe cytopenia was defined consistent with established hematologic criteria as hemoglobin <80 g/L, absolute neutrophil count <0.50 x 10^9/L, or platelet count <25 x 10^9/L (7, 8).

Adverse disease-specific clinical outcomes in LGLL analyzed were initiation of immunosuppressive therapy (IST), transfusion dependency, splenectomy, hematopoietic stem cell transplant, and death. Cytopenia response to treatment was evaluated based on periodical clinical and laboratory examinations after at least 3 months of therapy, using ECOG5998 criteria(9). Adverse disease-specific clinical outcomes of patients diagnosed with PCD included the presence of CRAB signs of symptomatic MM/lymphoma, and other paraneoplastic manifestations attributable to the M protein that in the absence of >10% BM plasma cell infiltration would be indicative of a monoclonal gammopathy of clinical significance (MGCS) (10).

Laboratory data at diagnosis complete blood count (CBC), BM exam, and routine tests performed in the clinic

as part of the diagnostic workup of LGLL (LGL count, lymphocyte subset characterization by flow cytometry, TCR rearrangement for T-cell clonality, *STAT3* mutational status) and PCDs (Ig levels, M protein assessment, myeloma FISH, IGH rearrangement for B-cell clonality, *MYD88* mutational status). Lymphocyte subset characterization by flow cytometry (in $CD45^{Ly}$ gated cells) routinely performed in the clinic in peripheral blood at LGLL diagnosis was also collected for the study of the following populations: T cells (CD3+, normal range [NR]: 0.96 - 2.39 $x10^9/L$), CD4+ T helper cells (CD3+CD4+, NR: 0.53 - 1.67 $x10^9/L$), CD8+ cytotoxic T lymphocytes (CTLs, CD3+CD8+, NR: 0.28 - 0.96 $x10^9/L$), NK cells (CD3-CD16/CD56+, NR: 0.10 - 0.57 $x10^9/L$) and B cells (CD19+, NR: 0.08 - 0.66 $x10^9/L$). The Ig levels analyzed included the quantification of IgG (NR: 717 - 1411 mg/dL), IgA (NR: 78 - 391 mg/dL) and IgM (NR: 53 - 334 mg/dL).

Serum levels of the proinflammatory cytokines IL1β (NR: <=6.7 pg/mL), IL2 (NR: <=2.1 pg/mL), sIL2R/sCD25 (NR: 175.3 - 858.2 pg/mL), IL4 (NR: <=2.2 pg/mL), IL5 (NR: <=2.1 pg/mL), IL6 (NR: <=2.0 pg/mL), IL8 (NR: <=3.0 pg/mL), IL10 (NR: <=2.8 pg/mL), IL12 (NR: <=1.9 pg/mL), IL13 (NR: <=2.3 pg/mL), IL17 (NR: <=1.4 pg/mL), TNFα (NR: <=7.2 pg/mL) and IFNγ (NR: <=4.2 pg/mL) were measured by quantitative multiplex bead assays performed in clinic routine laboratory.

Next generation sequencing

All patients were deep sequenced for the presence of a mutation in exon 21 of *STAT3*, the protein-protein interaction domain as previously described(11).

Targeted sequencing was performed as previously described using a custom panel for detection of hematological neoplasm gene variants from TruSeq or Nextera platforms (Illumina, San Diego, CA)(12, 13). A customized bioinformatic analytical pipeline was used to map reads to human genome hg38. During internal validation, this test delivered an average of >500X coverage and >98% of targeted regions showed over 100X coverage. The test demonstrated 95.2% sensitivity and 99.9% specificity in identifying single nucleotide variants (SNVs), small insertions and deletions (indels) (<10bp) of >5% variant allele fraction (VAF). For the identification of large indels (>10bp) >5% VAF, including FLT3 ITD, the test demonstrated 87.5% sensitivity and 99.9% specificity. The limit of detection of this test is 1% for the JAK2 V617F and NPM1 W288Cfs*12 variants, and 5% for other variants.

Whole-exome sequencing

Whole exome sequencing (WES) was performed by Novogene (Sacramento, CA) in genomic DNA (gDNA) extracted from peripheral blood mononuclear cells. Briefly, the gDNA was randomly sheared into short fragments (180-280 bp). The obtained fragments were end repaired, A-tailed, and further ligated with Illumina adapters. The fragments with adapters were PCR amplified, size selected, and purified. The prepped libraries were hybridized in the buffer with biotin-labeled probes, and magnetic beads with streptavidin were used to capture the exons of genes. Subsequently, non-hybridized fragments were washed out and probes were digested. The captured libraries were enriched by PCR amplification. The library was checked with Qubit and real-time PCR for quantification and bioanalyzer for size distribution detection. Quantified libraries were pooled and sequenced on Illumina platforms with PE150 strategy, according to effective library concentration

and data amount required.

Raw read files were first converted to FASTQ format, then aligned to human genome hg38 using the Burrows-Wheeler Aligner (BWA) (14). Aligned reads were processed using Genome Analysis Toolkit (GATK) that also extracted candidate variants/ polymorphisms to reduce sequencing errors (15).

Somatic variants filtering

We screened this genomic cohort for the presence of somatic variants in a list of 80 clonal hematopoiesis (CH)-related genes (**Supplemental Table 1**)(16). Detected variants were filtered using the following criteria: (i) only variants with a minimum depth of 10 reads and 4 reads supporting the alternate allele were considered; (ii) synonymous, polymorphisms (global population frequency >1%), and potential germline variants were removed; (iii) nonsynonymous variants (missense, nonsense, frameshift, and indels) were included, and further filtered according to mutational databases including COSMIC(17), ClinVar(18) and VarSome(19) for pathogenicity confirmation. Only pathogenic/likely pathogenic variants registered in COSMIC as canonical/recurrent and ever reported as somatic were selected to increase stringency in terms of clinical consequences.

Bone marrow cultures

Healthy donors undergoing harvest of BM hematopoietic stem and progenitor cells gave written informed consent for the collection of surplus cells purged from the filtration system. Erythroid and granulocytic cultures for detection and quantification of hematopoietic progenitor cells in colony-forming unit (CFU) assays were established in MethoCult™ methylcellulose-based media (STEMCELL™ Technologies, Vancouver, Canada) with addition of either patient serum or normal pooled serum from type AB healthy donor volunteers (MP Biochemicals, Solon, OH) at a final concentration of 20%. The count of erythroid and granulocytic CFUs (CFU-E and CFU-GM, respectively) was assessed on day 14. Each serum sample was assessed per duplicate-triplicate. Given inter-individual heterogeneity on the CFU potential of the different donor BM cells used, normalized CFU-E and -GM counts (% of total CFU as compared to the experiment control) were estimated.

Ethical approval

This research was approved by the institutional review board of Cleveland Clinic. Patient enrollment into clinical registry and specimen collection were performed in accordance with IRB 15-1278 and IRB 5024, respectively. The study was performed in accordance with the Declaration of Helsinki. Written informed consent was obtained from all participants.

Statistical analysis

Categorical variables were presented as percentages and compared using Pearson's chi-squared and Fisher’s exact tests. Continuous variables were presented as mean and SD if normally distributed, and as median and IQR if non-normally distributed. Differential analysis of categorical variables included Pearson’s

$\chi 2$ or Fisher's exact tests; comparison of continuous variables included Student's t test or nonparametric Mann-Whitney U test. Differences with a 2-tailed p-values less than 0.05 were considered statistically significant. Survival analysis between groups was done with Log-Rank test. Associations between clinical data and survival outcomes were assessed with unadjusted (univariable) and adjusted (multivariable) Cox regression. Overall survival Estimations were presented with 95% confidence intervals (95%CI).

To identify patterns of coordinated cytokine expression, unsupervised hierarchical clustering was performed using Euclidean distance as the similarity metric and Ward's minimum variance method (ward.D2) for agglomeration. Clustering was applied to both log10-transformed cytokine levels (columns) and patients (rows). Cluster assignment was subsequently used for downstream analyses, including the comparison of clinical, hematologic, and molecular features between groups. Comparisons between clusters were performed using Wilcoxon rank-sum test for continuous variables, and Chi-square or Fisher's exact tests, as appropriate, for categorical variables.

Gene pathway analysis was performed with using GeneMANIA (University of Toronto, Canada)(20) and Cytoscape (NIH, Bethesda, MD) (21).

Statistical analysis and graphic representation was performed using GraphPad Prism v.9.4 (GraphPad Software Inc., San Diego, CA) and STATA v.16 (StataCorp LLC, College Station, TX), or R (R Core Team, Vienna, Austria)(22).

Supplemental Tables

Supplemental Table 1 (Excel file). Panel of clonal hemopoiesis-related genes screened in this study. Ensembl annotations are provided.

Supplemental Table 2 (Excel file). Somatic mutations in clonal hematopoiesis-related genes. Information regarding variant cDNA and protein changes (hg38), and VAF is provided. Abbreviations: n: number of cases.

Supplemental Table 3 (Excel file). Multivariable logistic regression on the risk of cytopenias in PCDs. †Definitions of cytopenia of ant type and severity: Hgb < 12 g/dl (13 g/dl in men), Neu < 1.5 x109/L, Ptl < 100 x109/L. Definition of severe cytopenia of any type: Hgb < 8 g/dL, Neu < 0.5 x109/L, Ptl < 25 x109/L. Definitions of immune-mediated cytopenia: AIHA, AIN, ITP, AA, PRCA. Hgb: hemoglobin. Neu: neutrophils. Ptl: platelets. AIHA: autoimmune hemolytic anemia. AIN: autoimmune neutropenia. ITP: primary immune thrombocytopenia. AA: aplastic anemia. PRCA: pure red cell aplasia.

Supplemental Table 4 (Excel file). Multivariable logistic regression on the risk of cytopenias in LGLL. †Definitions of cytopenia of ant type and severity: Hgb < 12 g/dl (13 g/dl in men), Neu < 1.5 x109/L, Ptl < 100 x109/L. Definition of severe cytopenia of any type: Hgb < 8 g/dL, Neu < 0.5 x109/L, Ptl < 25 x109/L. Definitions of immune-mediated cytopenia: AIHA, AIN, ITP, AA, PRCA. Hgb: hemoglobin. Neu: neutrophils. Ptl: platelets. AIHA: autoimmune hemolytic anemia. AIN: autoimmune neutropenia. ITP: primary immune thrombocytopenia. AA: aplastic anemia. PRCA: pure red cell aplasia.

Supplemental Table 5 (Excel file). Multivariable regression analysis of LGLL-specific adverse outcomes. †Competitive risk regression analysis (death was treated as competitive event).

Supplemental Table 6 (Excel file). Multivariable regression analysis of PCD-specific adverse outcomes. †Competitive risk regression analysis (death was treated as competitive event).

Supplemental Table 7 (Excel file). Clinical and laboratory features of LGLL patients with extended panel of serum proinflammatory cytokines and genomic data for unsupervised clustering (n=22). BCN: B-cell neoplasm, CH: clonal hematopoiesis, PCD: plasma cell dyscrasia.

Supplemental Figures

Supplemental Figure 1. Summary of study design. A) Summary of patient flow. B) Detailed summary of patient flow in strategy no. 1. Abbreviations: BM: bone marrow exam. LGLL: large granular lymphocyte leukemia. MPA: M protein assessment. PCD: plasma cell dyscrasia.

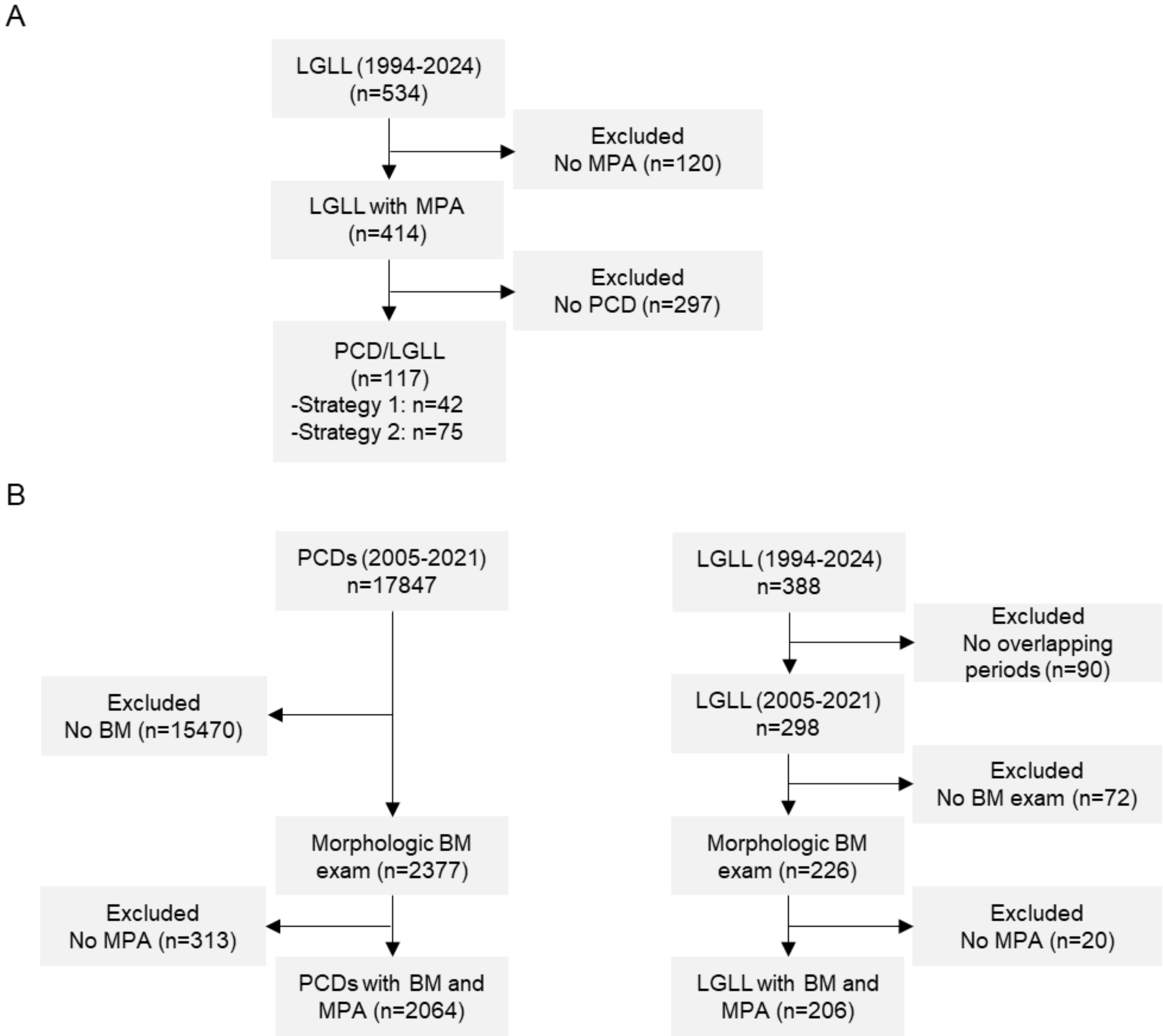

Supplemental Figure 2. Overall survival analysis. Abbreviations: PCD: plasma cell dyscrasia. LGLL: large granular lymphocyte leukemia. PCD/LGLL: PCD/LGLL dyad. Log-rank p = ns.

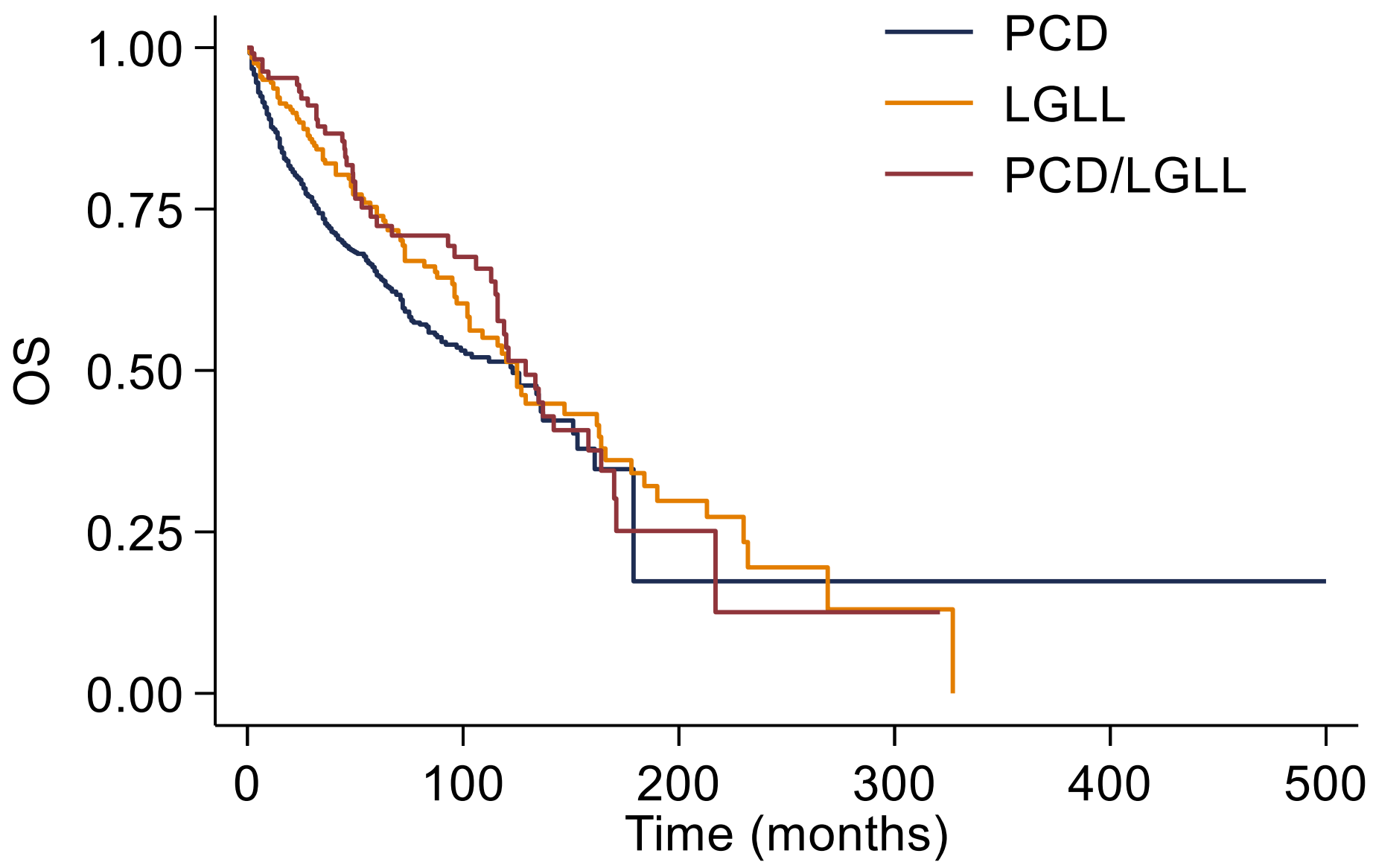

No. at risk

	0	100	200	300	400	500
PCD	671	109	1	1	1	1
LGLL	248	58	13	1	0	0
PCD/LGLL	113	38	3	1	0	0

Supplemental Figure 3. Cytokine clustering in T-LGLL patients (n=22). A) Unsupervised hierarchical clustering of serum cytokine levels. Clustering was performed using Euclidean distance and Ward's linkage method, identifying two major patient groups with distinct serum inflammatory signatures: cluster 1 (high levels) and cluster 2 (lower levels). **B) Distribution of key features across cytokine-defined clusters.** Bar plots depict the proportion of patients presenting selected key features in cluster 1 (red) vs. cluster 2 (blue). **C) Serum cytokine levels according to cluster assignment.** Box plots show the distribution of individual cytokines in cluster 1 (red) vs. cluster 2 (blue). Comparisons were performed using the Wilcoxon rank-sum test. Only statistically significant differences are shown. *p < 0.05, **p < 0.01, and ***p < 0.001. Abbreviations: BCN: B-cell neoplasms. CH: clonal hematopoiesis, IL: interleukin. IL2R: soluble IL-2 receptor. IFN: interferon gamma. PCD: plasma cell dyscrasias. TNF: tumor necrosis factor alpha.

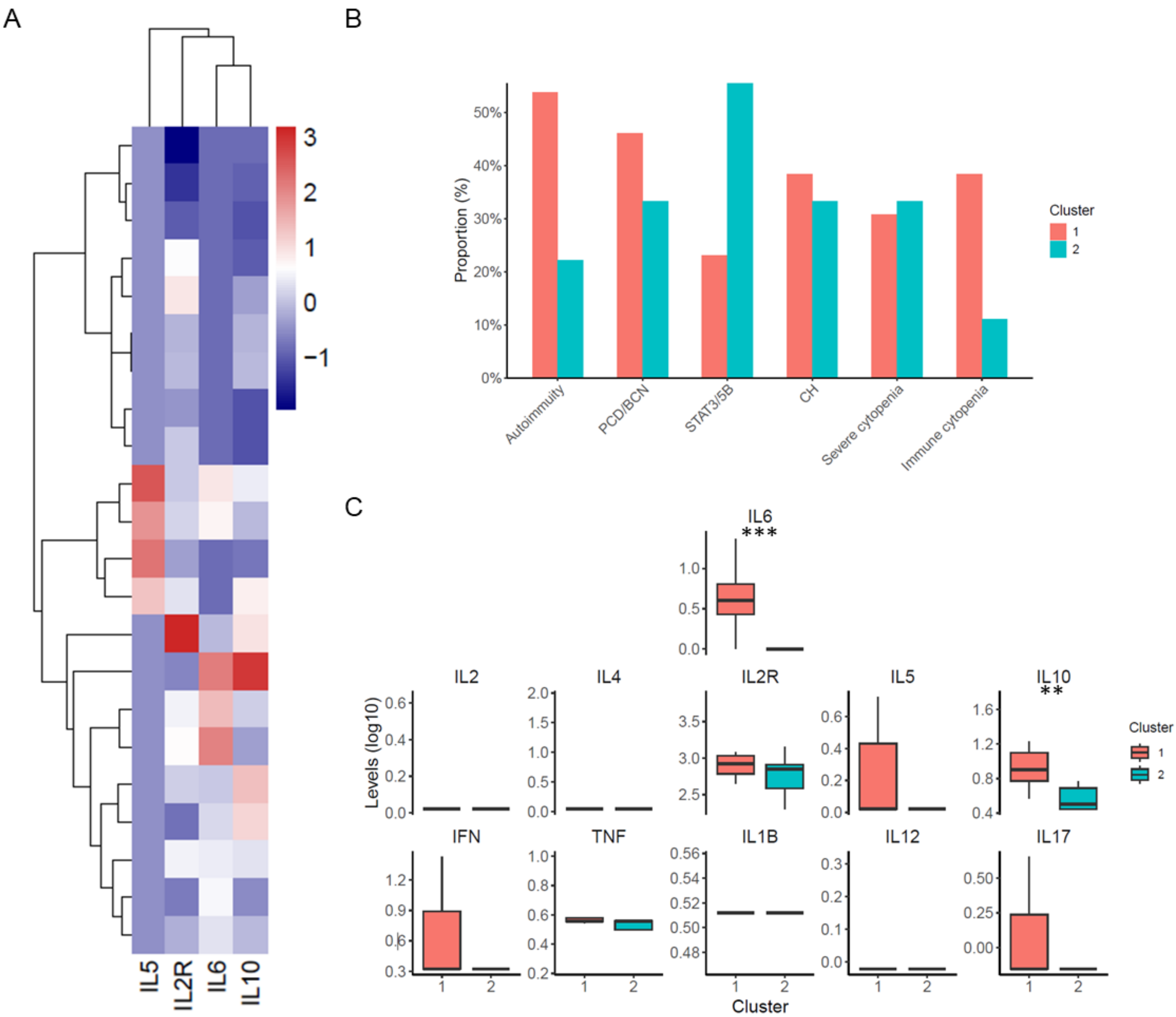

Supplemental Figure 4. Extended panel of serum proinflammatory cytokines in LGLL patients (n=26). The reference lines represent the upper limit of normality. Abbreviations: IL: interleukin. IFNγ: interferon gamma. TNFα: tumor necrosis factor alpha. sIL-2R: soluble IL-2 receptor. MGUS: monoclonal gammopathy of unknown significance.

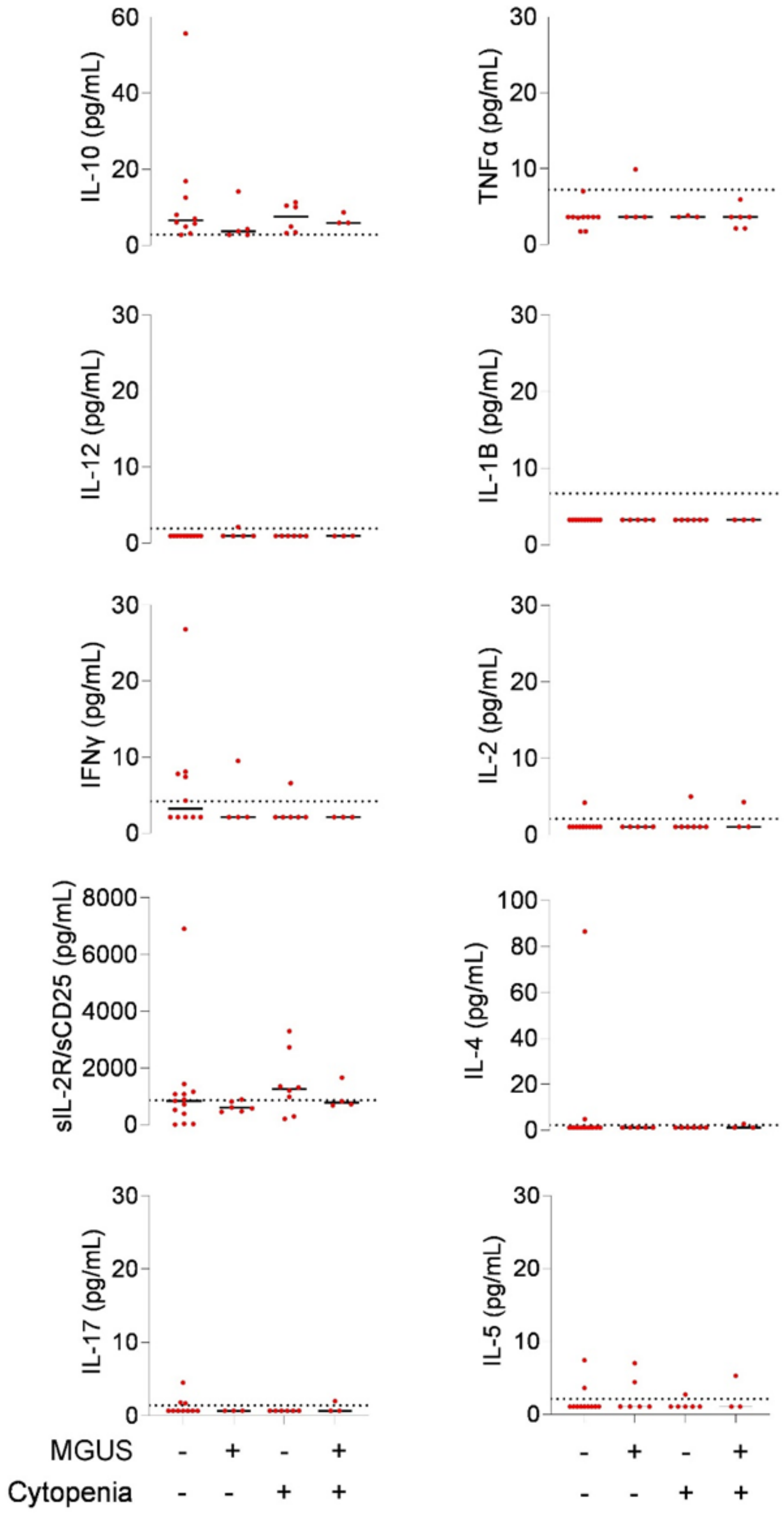

References

1. Zawit M, et al. Large Granular Lymphocytic Leukemia: From Immunopathogenesis to Treatment of Refractory Disease. *Cancers (Basel)*. 2021;13(17). https://doi.org/10.3390/cancers13174418.
2. Alaggio R, et al. The 5th edition of the World Health Organization Classification of Haematolymphoid Tumours: Lymphoid Neoplasms. *Leukemia*. 2022;36(7):1720–1748.
3. Treon SP, et al. Report of consensus Panel 4 from the 11th International Workshop on Waldenstrom's macroglobulinemia on diagnostic and response criteria. *Semin Hematol*. 2023;60(2):97–106.
4. Rajkumar SV, et al. International Myeloma Working Group updated criteria for the diagnosis of multiple myeloma. *Lancet Oncol*. 2014;15(12):e538-548.
5. Murray DL, et al. Laboratory persistence and clinical progression of small monoclonal abnormalities. *Am J Clin Pathol*. 2012;138(4):609–613.
6. Gurnari C, et al. Monoclonal IgM gammopathy in adult acquired pure red cell aplasia: culprit or innocent bystander? *Blood Cells Mol Dis*. 2021;91:102595.
7. World Health Organization. Guideline on haemoglobin cutoffs to define anaemia in individuals and populations. Geneva: WHO. 2024. https://www.who.int/publications/i/item/9789240088542.
8. U.S. Department of Health and Human Services. Common Terminology Criteria for Adverse Events (CTCAE) Version 5.0. National Cancer Institute. 2017. https://dctd.cancer.gov/research/ctep-trials/for-sites/adverse-events/ctcae-v5-5x7.pdf.
9. Poullot E, et al. Chronic natural killer lymphoproliferative disorders: characteristics of an international cohort of 70 patients. *Ann Oncol*. 2014;25(10):2030–2035.
10. Dispenzieri A. Monoclonal gammopathies of clinical significance. *Hematology Am Soc Hematol Educ Program*. 2020;2020(1):380–388.
11. Andersson E, et al. Activating somatic mutations outside the SH2-domain of STAT3 in LGL leukemia. *Leukemia*. 2016;30(5):1204–1208.
12. Gurnari C, et al. A study of Telomerase Reverse Transcriptase rare variants in myeloid neoplasia. *Hematol Oncol*. 2022;40(4):812–817.
13. Adema V, et al. Pathophysiologic and clinical implications of molecular profiles resultant from deletion 5q. *EBioMedicine*. 2022;80:104059.
14. Li H, Durbin R. Fast and accurate long-read alignment with Burrows-Wheeler transform. *Bioinformatics*. 2010;26(5):589–595.
15. McKenna A, et al. The Genome Analysis Toolkit: a MapReduce framework for analyzing next-generation DNA sequencing data. *Genome Res*. 2010;20(9):1297–1303.
16. Niroula A, et al. Distinction of lymphoid and myeloid clonal hematopoiesis. *Nat Med*. 2021;27(11):1921–1927.
17. Tate JG, et al. COSMIC: the Catalogue Of Somatic Mutations In Cancer. *Nucleic Acids Res*. 2019;47(D1):D941–D947.
18. Landrum MJ, et al. ClinVar: improving access to variant interpretations and supporting evidence. *Nucleic Acids Res*. 2018;46(D1):D1062–D1067.
19. Kopanos C, et al. VarSome: the human genomic variant search engine. *Bioinformatics*. 2019;35(11):1978–1980.
20. Warde-Farley D, et al. The GeneMANIA prediction server: biological network integration for gene prioritization and predicting gene function. *Nucleic Acids Res*. 2010;38(Web Server issue):W214-220.
21. Shannon P, et al. Cytoscape: a software environment for integrated models of biomolecular interaction networks. *Genome Res*. 2003;13(11):2498–2504.
22. R Core Team. R: A Language and Environment for Statistical Computing. 2018. https://www.R-project.org/.
